# Inhibition of Oral Pathogenic Bacteria, Suppression of Bacterial Adhesion and Invasion on Human Squamous Carcinoma Cell Line (HSC-4 Cells), and Antioxidant Activity of Plant Extracts from Acanthaceae Family

**DOI:** 10.3390/plants13182622

**Published:** 2024-09-20

**Authors:** Sureeporn Suriyaprom, Pornpimon Ngamsaard, Varachaya Intachaisri, Nitsanat Cheepchirasuk, Aussara Panya, Thida Kaewkod, Yingmanee Tragoolpua

**Affiliations:** 1 Department of Biology, Faculty of Science, Chiang Mai University, Chiang Mai 50200, Thailand; sureeporn.suriyaprom@cmu.ac.th (S.S.); promjinnapimon@gmail.com (P.N.); warachaya_khing@hotmail.com (V.I.); nitsanat_cheep@cmu.ac.th (N.C.); aussara.pan@cmu.ac.th (A.P.); thida.kaewkod@cmu.ac.th (T.K.); 2 Office of Research Administration, Chiang Mai University, Chiang Mai 50200, Thailand; 3 Natural Extracts and Innovative Products for Alternative Healthcare Research Group, Faculty of Science, Chiang Mai University, Chiang Mai 50200, Thailand

**Keywords:** Acanthaceae family plant, antioxidant, biofilm, oral pathogenic bacteria, *Streptococcus mutans*

## Abstract

Medicinal plants have traditionally been used to treat various human diseases worldwide. In this study, we evaluated the leaf extracts of plants from the Acanthaceae family, specifically *Clinacanthus nutans* (Burm.f.) Lindau, *Thunbergia laurifolia* Lindl., and *Acanthus ebracteatus* Vahl., for their compounds and antioxidant activity. The ethanolic extracts of *A. ebracteatus* showed the highest total phenolic content at 22.55 mg GAE/g extract and the strongest antioxidant activities, with IC_50_ values of 0.24 mg/mL and 3.05 mg/mL, as determined by DPPH and ABTS assays. The antibacterial efficacy of these extracts was also tested against *Streptococcus pyogenes*, *Streptococcus mutans*, *Staphylococcus aureus*, and *Klebsiella pneumoniae*. The diameters of the inhibition zones ranged from 14.7 to 17.3 mm using the agar well diffusion method, with MIC and MBC values ranging from 7.81 to 250 mg/mL. Anti-biofilm formation, antibacterial adhesion, and antibacterial invasion assays further demonstrated that these medicinal plant extracts can inhibit bacterial biofilm formation and prevent the adhesion and invasion of oral pathogenic bacteria on the human tongue squamous cell carcinoma-derived cell line (HSC-4 cells). The ethanolic extracts of *C. nutans* and *A. ebracteatus* were able to inhibit the *gtfD* and *gbp* genes, which facilitate biofilm formation and bacterial adherence to surfaces. These findings provide new insights into the antibacterial and antioxidant properties of plant extracts from the Acanthaceae family. These activities could enhance the clinical and pharmaceutical applications of plant extracts as an alternative therapy for bacterial infections and a dietary supplement.

## 1. Introduction

The 2022 WHO Global Oral Health Status Report estimated that oral diseases affect nearly 3.5 billion people worldwide, despite being largely preventable. These diseases pose a significant global health burden and are increasingly linked to various systemic conditions, such as infective endocarditis and respiratory infections [1,2]. Oral infections that are caused by the colonization of numerous microorganisms [3] can contribute to chronic diseases. *Streptococcus mutans* is particularly implicated in dental caries through biofilm formation [4]. Although antibacterial agents such as sodium hypochlorite and chlorhexidine are commonly used, they can have several adverse effects, such as tooth discoloration and drug sensitivity [4]. Moreover, a major concern is the rising resistance to conventional antibiotics. Therefore, the prevention of oral pathogenic bacterial infection is crucial in managing oral health.

Medicinal plants have long been used to treat various diseases. The Acanthaceae family is notable for its pharmacological potential. Phytochemicals in this family include glycosides, flavonoids, and phenolic compounds [5]. Among these, *Clinacanthus nutans* (Burm.f.) Lindau (sabah snake grass) is well known in Southeast Asia for its antioxidant, anti-inflammatory, antiviral, and antimicrobial activities. It has been traditionally used to treat conditions like skin rashes, diabetes, and cancer, and also inhibits the growth of harmful bacteria such as *Bacillus cereus* and *Escherichia coli* [6,7]. Similarly, *Thunbergia laurifolia* Lindl. (blue trumpet vine) contains flavonoids and phenolic acids and is traditionally used as an antidote for poisons [8]. It also exhibits antioxidant, antimicrobial, and anti-inflammatory properties [9,10,11]. *Acanthus ebracteatus* Vahl. (sea holly), another plant in this family, is used in Southeast Asia for its anti-inflammatory and wound-healing effects. It has been found to inhibit bacterial growth, including *Staphylococcus aureus* and *Klebsiella pneumoniae* [12,13,14,15,16]. These plants highlight the medicinal importance of the Acanthaceae family and their potential for further therapeutic applications. With a wide range of pharmacological properties, they offer significant potential for the development of modern therapeutic agents. Their diverse bioactive compounds make them promising candidates for further research into treatments of infectious diseases, inflammation, and other health conditions.

Therefore, we have evaluated the compounds and antioxidant activity of plant extracts from the Acanthaceae family, focusing on *Clinacanthus nutans* (Burm.f.) Lindau, *Thunbergia laurifolia* Lindl., and *Acanthus ebracteatus* Vahl. in this study. Antibacterial activities against oral pathogenic bacteria, including *Streptococcus pyogenes*, *Streptococcus mutans, Staphylococcus aureus*, and *Klebsiella pneumoniae,* were investigated. Moreover, the abilities of these plant extracts to inhibit bacterial growth, prevent biofilm formation, and affect bacterial adhesion and invasion on oral squamous cells were examined. Additionally, the regulations of virulence genes in *S. mutans* that contributed to biofilm formation and bacterial adhesion were examined.

## 2. Results

### 2.1. Total Phenolic, Total Flavonoid, and Antioxidant Activity of Acanthaceae Family Plant Extracts

Plant extracts from the Acanthaceae family were assessed through measurements of total phenolic and flavonoid contents, as well as antioxidant activity. The ethanolic extract of *A. ebracteatus* had the highest total phenolic content at 22.55 mg gallic acid equivalent per gram extract (mg GAE/g extract), while *C. nutans* had the lowest at 1.86 mg GAE/g extract. For flavonoid content, *C. nutans* exhibited the highest level at 14.12 mg quercetin equivalent per gram extract (mg QE/g extract), whereas *T. laurifolia* had the lowest at 3.29 mg QE/g extract (Table 1).

The antioxidant activity of plant extracts was measured (Table 2). The DPPH radical scavenging activity and ABTS radical cation decolorization activity of plant extracts were expressed as IC_50_ values, indicating the concentration of each extract needed to inhibit 50% of the initial free radicals. *A. ebracteatus* extract showed the most potent DPPH radical scavenging activity with an IC_50_ of 0.24 mg/mL and the highest ABTS radical cation decolorization activity with an IC_50_ of 3.05 mg/mL. In terms of antioxidant activity, *A. ebracteatus* also had the highest values for both assays, with 24.41 mg GAE/g extract for the DPPH assay and 57.47 mg Trolox equivalent antioxidant capacity (TEAC) per gram of extract (mg TEAC/g extract) for the ABTS assay. Conversely, *C. nutans* had the lowest DPPH antioxidant activity with 6.75 mg GAE/g extract and the lowest ABTS activity with 28.67 mg TEAC/g extract.

### 2.2. Antibacterial Activity of Plant Extracts from the Acanthaceae Family against Some Oral Pathogenic Bacteria by Agar Well Diffusion Method

**Figure 1:**
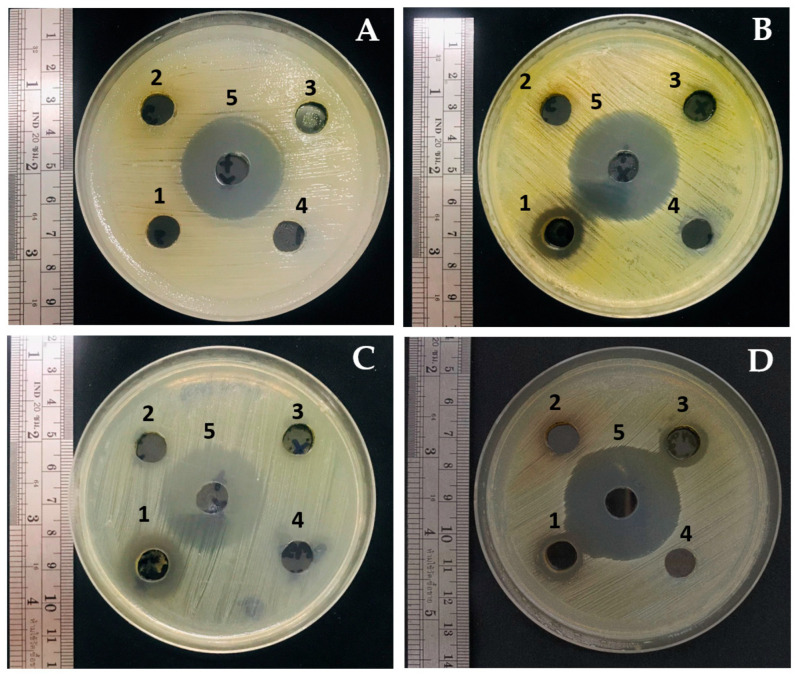
Effect of plant extracts from the Acanthaceae family against some oral pathogenic bacteria (**A**) *K. pneumoniae*, (**B**) *S. aureus*, (**C**) *S. mutans*, and (**D**) *S. pyogenes* by agar well diffusion method. Wells were loaded with the following treatments: (1) *T. laurifolia*, (2) *A. ebracteatus,* (3) *C. nutans*, (4) negative controls (DMSO 99.9%), and (5) positive controls (gentamicin 1 mg/mL).

The antibacterial activity of plant extracts against oral pathogenic bacteria, including *K. pneumoniae*, *S. aureus*, *S. mutans*, and *S. pyogenes*, was evaluated using the agar well diffusion method. The diameters of inhibition zones were measured to assess the effectiveness of extracts. Results revealed that extracts from *T. laurifolia* at 125 mg/mL effectively inhibited all tested bacteria except *K. pneumoniae*, with inhibition zone diameters ranging from 14.7 to 17.3 mm (Table 3 and Figure 1). The positive controls (gentamicin) exhibited inhibition zones of 24.3–27.3 mm, while no clear zones were observed for the negative controls (DMSO 99.9%). Additionally, the *C. nutans* extract demonstrated inhibitory effects on *S. pyogenes*, whereas the *A. ebracteatus* extract did not inhibit all tested bacteria.

### 2.3. Antibacterial Activity of Plant Extracts from the Acanthaceae Family as Determined by Minimum Inhibitory Concentration (MIC) and Minimum Bactericidal Concentration (MBC)

The minimum inhibitory concentration (MIC) and minimum bactericidal concentration (MBC) of the plant extracts were determined using the broth dilution method. Results indicated that the extracts were effective against all bacterial species tested, with MIC and MBC values ranging from 7.81 to 250 mg/mL (Table 4). *T. laurifolia* extract exhibited the highest activity against *S. aureus*, *S. mutans*, and *S. pyogenes*, with MIC and MBC values of 15.63, 15.63, and 7.81 mg/mL, respectively. In contrast, *A. ebracteatus* extract demonstrated the lowest activity against *S. pyogenes*, with MIC and MBC values of 250 mg/mL. Gentamicin, used as a positive control, effectively inhibited the growth of all tested bacteria, with MIC and MBC values ranging from 0.0039 to 0.125 mg/mL.

### 2.4. Antibacterial Activity of Plant Extracts from the Acanthaceae Family against Some Oral Pathogenic Bacteria by Time Kill Assay

**Figure 2:**
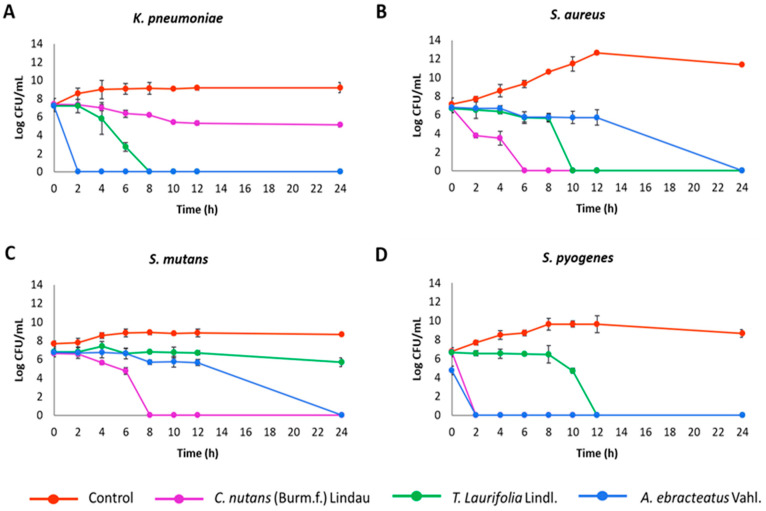
Effect of plant extracts from the Acanthaceae family (1 MIC) against (**A**) *K. pneumoniae*, (**B**) *S. aureus*, (**C**) *S. mutans*, and (**D**) *S. pyogenes*. Time–kill data represented as mean ± SD (n = 3).

The bactericidal efficacy of plant extracts from the Acanthaceae family was assessed by monitoring the time required to eradicate the tested bacteria. The duration to achieve no viable cell count varied among the bacterial species and the different plant extracts used (Figure 2 and Supplementary Materials Table S1). According to the results, the extracts from *A. ebracteatus* completely eradicated *K. pneumoniae* within 2 h, whereas the extract from *T. laurifolia* achieved the same result within 8 h. Additionally, the extracts from *C. nutans* and *T. laurifolia* completely inhibited *S. aureus* after 6 and 10 h of incubation, respectively. Furthermore, *C. nutans* extracts exhibited the strongest inhibitory effect against *S. mutans* at 8 h. For *S. pyogenes*, the extracts from *C. nutans* and *A. ebracteatus* were most effective, eliminating the bacteria within 2 h, while the extract from *T. laurifolia* achieved complete eradication within 12 h.

### 2.5. Antibacterial Activity of Plant Extracts from the Acanthaceae Family on Biofilm Formation of Oral Pathogenic Bacteria

**Figure 3:**
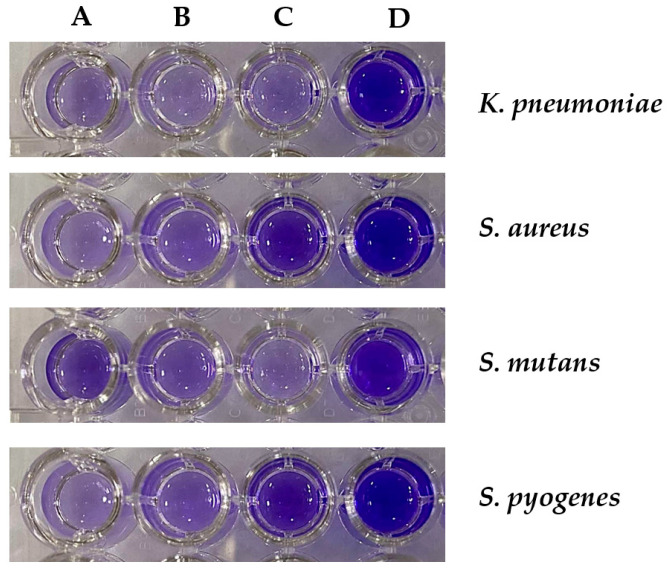
Effect of plant extracts from the Acanthaceae family on the biofilm formation of oral pathogenic bacteria: *K. pneumoniae*, *S. aureus*, *S. mutans*, and *S. pyogenes* after treatment with (**A**) *C. nutans*, (**B**) *T. laurifolia*, (**C**) *A. ebracteatus,* and (**D**) bacterial control group, which was not treated with the extracts.

The effects of plant extracts from the Acanthaceae family on the inhibition of biofilm formation against some oral pathogenic bacteria are demonstrated in Table 5 and Figure 3. The results were shown as percentages of biofilm inhibition comparing to the bacterial control group that was not treated with extracts, with inhibition ranging from 34.22% to 96.84%. Plant extracts from *C. nutans* were most effective against biofilm formation by *K. pneumoniae*, *S. aureus*, and *S. pyogenes*, with inhibition between 88.89 and 95.12%. Moreover, *A. ebracteatus* extract significantly inhibited biofilm formation in *S. mutans* and *S. pyogenes* by 95.45 and 96.84%. Conversely, *T. laurifolia* extract had the least effect on biofilm formation in *S. pyogenes* by 34.22%.

### 2.6. Antibacterial Activity of Plant Extracts from the Acanthaceae Family against Some Oral Pathogenic Bacteria by Adhesion and Invasion Assay

**Figure 4:**
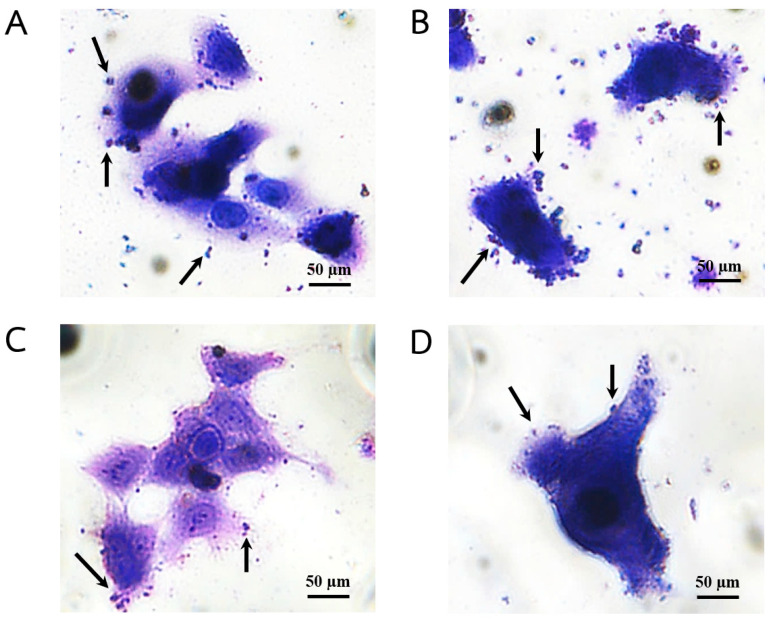
Effect of plant extracts from the Acanthaceae family on bacterial adhesion to HSC-4 cells. Adhesion of bacterial control group (**A**) *S. mutans* to HSC-4 cells and after being treated with (**B**) *C. nutans* (Burm.f.) Lindau, (**C**) *T. laurifolia* Lindl., and (**D**) *A. ebracteatus* Vahl. extracts as observed by microscopic examination. Arrows indicate representative adhered bacterial cell.

The antibacterial adhesion and invasion effects of plant extracts from the Acanthaceae family were assessed using human tongue squamous cell carcinoma-derived cell line (HSC-4 cells). The results were expressed as a percentage of antibacterial adhesion and invasion of *S. mutans* relative to the bacterial control group that was not treated with extracts. While the percentage of antibacterial adhesion activity ranged between 22.47 and 48.97% and antibacterial invasion activity ranged between 70.83 and 80.97% (Table 6), *T. laurifoli* extract was the most effective against *S. mutans* adhesion to HSC-4 cells (Figure 4), but in contrast, it showed the least activity in reducing *S. mutans* invasion. Additionally, extracts from *C. nutans* and *A. ebracteatus* were efficient against *S. mutans* invasion with inhibition rates of 80.40 and 80.97%.

### 2.7. Effect of Plant Extracts from the Acanthaceae Family on Gene Expression of S. mutans

**Figure 5:**
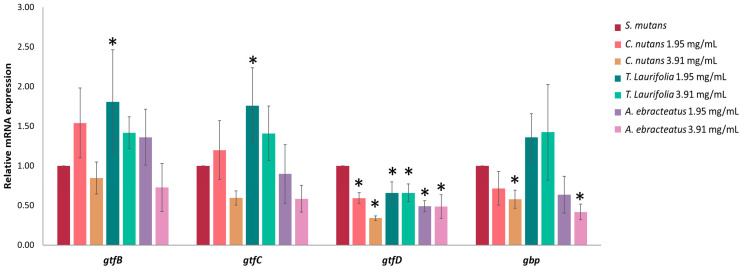
The relative mRNA expression levels of *gtfB, gtfC, gtfD,* and *gbp* genes on *S. mutans* after treatment with plant extracts from the Acanthaceae family at 1.95 and 3.91 mg/mL. Gene expression ratios of *S. mutans* were normalized and compared to the expression of the regulator gene. Data are expressed as mean ± SD (n = 3), and significant difference (*) with *p* < 0.05 when comparing to the untreated bacterial control group is indicated.

*S. mutans* produces glucosyltransferases (GTFs) to create glucans, which facilitate bacterial adhesion and biofilm formation by binding to the interface between bacteria and the surface. The mRNA expression of glucosyltransferases (*gtf*) and glucan-binding protein (*gbp*) genes was studied by real-time qPCR. The results showed that the effect of *C. nutans*, *T. laurifolia,* and *A. ebracteatus* extracts at concentrations of 1.95 and 3.91 mg/mL could significantly downregulate the *gtfD* gene when compared to the bacterial control group that was not treated with the extracts. Moreover, the extracts of *C. nutans* and *A. ebracteatus* at a concentration of 3.91 mg/mL significantly suppressed the mRNA expression of *gbp* and also inhibited the mRNA expression of the *gtfB* and *gtfC* gene without significant differences compared to the bacterial control group that was not treated with extracts. However, the extracts of *T. laurifolia* did not downregulate the mRNA expression levels *gtfB* and *gtfC* (*p* < 0.05) in the same conditions (Figure 5).

## 3. Discussion

The Acanthaceae family encompasses a wide variety of plants known for their significant medicinal properties, which are attributed to their abundant phytochemical content. Extracts from *C. nutans*, specifically from northern Thailand, have been extensively analyzed using techniques such as TLC, HPLC-UV/DAD, LC-MS/MS [17], and GC-MS/MS [18]. The studies identified several bioactive compounds, including glyceryl 1,3-distearate, a glycolipid, and kaempferol 3-O-feruloyl-sophoroside 7-O-glucoside, a C-glycoside flavonoid. Similarly, the leaf extract of *T. laurifolia*, sourced from Thailand, has been extensively analyzed using HPLC methods, revealing key bioactive compounds such as caffeic acid, vitexin, and rosmarinic acid [19,20]. Furthermore, the leaf extract of *A. ebracteatus*, also collected in Thailand, was subjected to UHPLC-QTOF-MS analysis, which identified a range of bioactive constituents, including flavonoids, phenols, iridoids, and nucleosides [10].

This study found that the total phenolic and flavonoid content in plant extracts from the Acanthaceae family varied widely. Notably, the extract of *A. ebracteatus* exhibited a high concentration of phenolic compounds and demonstrated substantial antioxidant activity in both DPPH and ABTS assays. However, the total phenolic and flavonoid contents observed in our study differed from those reported in previous studies [12,13,21]. These variations may be due to differences in plant origin, growth conditions, extraction methods, and solvents used [21]. Significant attention has been directed towards the antioxidant and therapeutic properties of various medicinal plants. The high antioxidant capacity of the extract in this study can be attributed to its high total phenolic content, as many phenolic compounds are capable of scavenging free radicals in numerous plant species, including *A. ebracteatus* [21]. Although flavonoids presented in smaller quantities in the extract, their contribution to antioxidant activity has also been documented [22].

Using plant extracts with medicinal properties offers a viable alternative for treating various pathological conditions. Many plant extracts and natural products have demonstrated antimicrobial properties, contributing to the development of new drugs that can significantly improve the management of numerous health disorders [12]. Given that *S. mutans* is a primary causative agent of oral disease, we chose this bacterium for our study, along with other important oral bacteria such as *S. pyogenes*, *S. aureus*, and *K. pneumoniae*. The antibacterial activity of medicinal plants varies depending on the bacterial strain and the specific extracts used, which demonstrated positive antibacterial effects in both the agar well diffusion and broth dilution methods. The ethanolic extract of *T. laurifolia* at a concentration of 125 mg/mL was the most effective, showing a higher bactericidal effect against all tested strains, except *K. pneumoniae* by the agar well diffusion method. The positive control, gentamicin at 1 mg/mL, inhibited all tested bacteria. Gentamicin is a well-known antibiotic that specifically inhibits bacterial growth by targeting protein biosynthesis [23]. However, plant extracts often have broader and less targeted mechanisms of action. As a result, higher concentrations of plant extracts are typically required to achieve similar levels of bacterial inhibition. This is because plant extracts contain a complex mixture of bioactive compounds and each compound has varying effects on bacterial cells that make them less potent on antibacterial activity when compared on a per-milligram basis to the highly targeted and concentrated effects of pure gentamicin compound [24].

However, the exact mechanism of action of the extracts on microorganisms remains uncertain until the specific compound responsible for their antimicrobial effect is identified, but it can be estimated based on the general cell disruption effects, which are commonly associated with phenolic and flavonoid compounds found in plants of the Acanthaceae family [5]. The Acanthaceae family includes a diverse range of plants renowned for their abundant phytochemical content, particularly phenolic compounds and flavonoids. These bioactive substances are highly valued for their strong antioxidant effects and significant antibacterial properties. The antibacterial effectiveness of these plants is largely due to their high concentrations of phenolic and flavonoid compounds, which can damage bacterial cell walls, impede protein synthesis, and disrupt critical metabolic pathways within the bacteria [5].

Bacterial susceptibility to phytochemicals varies depending on the structure of their cell envelope, differing among bacteria. Gram-positive bacteria are more sensitive to antimicrobial agents due to their thick peptidoglycan layer, while Gram-negative bacteria have a thin peptidoglycan layer and an outer lipopolysaccharide membrane [25]. The presence of a thick peptidoglycan layer in Gram-positive bacteria facilitates the action of antimicrobial agents. Phenolic compounds, with their partial hydrophobic nature, are effective antibacterial agents because they can interact with the lipid–water interface of the bacterial membrane. This interaction can significantly reduce the plasticity of the membrane, leading to its destabilization. This weakening of the membrane integrity may result in the disruption of the bacterial membrane and critical transport processes [26]. According to the results, some medicinal plant extracts did not show an inhibition zone using the agar well diffusion method but still exhibited activity against the tested bacteria with the broth dilution method. The agar well diffusion method has been described as having relatively low sensitivity because the samples become further diluted as they diffuse into the agar [27]. However, the broth dilution methods, MIC and MBC, overcome some limitations of the agar diffusion method by allowing the extracts to make direct contact with the bacterial cells, proving to be more effective. Therefore, combining the agar well diffusion method with other procedures, such as the broth dilution method, is recommended for antimicrobial susceptibility testing [28]. Meanwhile, the time–kill analysis revealed varying degrees of time-dependent microbial inhibition among different bacteria and plant extracts. Antibacterial properties can be attributed to plant secondary metabolites and their response to microbial infection [29].

Biofilm formation has been documented in various bacterial species. It begins with the attachment of planktonic cells to a surface, progressing to an irreversible attachment [30]. Biofilms are significantly more resistant to antimicrobial agents than planktonic cells. In this study, plant extracts from the Acanthaceae family were able to inhibit the biofilm formation of oral pathogenic bacteria, with inhibition rates ranging from 34.22% to 96.84%. Previous studies have shown that sub-MIC levels of *C. nutans* can inhibit the bacterial attachment and biofilm formation of *S. mutans*. At sub-MIC levels, potential antimicrobial agents inhibited the sucrose-dependent initial attachment of *S. mutans*, preventing subsequent mature biofilm formation. Although the mechanisms behind the inhibitory effect of these medicinal plant extracts on biofilm formation were not fully understood, their anti-biofilm activity might be related to factors such as acid tolerance, water-insoluble glucan formation, and sucrose-dependent adherence [31]. Acanthaceae family plants are effective against bacterial infections and significantly impact the biofilm formation of *Pseudomonas aeruginosa*, *S. aureus*, and *E. coli* [32]. It reduces the production of extracellular pathogenic factors in *P. aeruginosa*, which are regulated by the quorum sensing (QS) system. Their active component, i.e., andrographolide, inhibits biofilm formation by targeting the QS system and regulating the staphylococcal accessory regulator A (SarA) factor in *S. aureus* [33]. Additionally, andrographolide reduces *E. coli* adhesion by decreasing the production of polysaccharide intercellular adhesin and poly-N-acetylglucosamine (PIA/PNAG), which is crucial for biofilm formation [34]. Natural substances, e.g., flavonoids, terpenoids, lectins, alkaloids, polypeptides, polyacetylenes, and phenolics, act on biofilms through various mechanisms. These mechanisms include restricting substrates, rupturing cell walls, affecting adhesion groups and cellular membranes, binding to proteins, and associating with DNA, and ultimately blocking viral fusion [35]. Moreover, DMSO that is used to dissolve the extracts might have inhibitory activity against biofilm formation in some bacteria [36].

The focus on HSC-4 cells adds a unique dimension to this study by examining how plant extracts from the Acanthaceae family affect bacterial interactions at a cellular level and closely mimic human oral tissues. The ability to adhere to and invade host cells is a crucial virulence factor for many pathogenic bacteria. This dynamic interaction between the bacterium and the host cell can be studied by analyzing the adherence and entry of bacteria into cultured mammalian cells [37]. In our study, the antibacterial adhesion activity of Acanthaceae plant extracts to oral squamous cells varied from 22.47% to 48.97%, while the antibacterial invasion activity of *S. mutans* ranged from 70.83% to 80.97%. These findings suggest that the plant extracts not only inhibit bacterial growth but also reduce bacterial adhesion and invasion at the cellular level, highlighting their potential role in modulating host–pathogen interactions and preventing bacterial colonization in oral tissues. Some herbal extracts, e.g., *A. paniculate*, *Streblus asper*, *Harrisonia perforate*, and *Cassia alata*, were able to inhibit the adherence of *S. mutans* by at least 50% [38]. *S. mutans* can produce firmly bound glucan from sucrose through the cooperative actions of glucosyltransferase (GTF). Studies on the mechanism of action showed that GTF activity was reduced by some herbal extracts, indicating that these extracts might interfere with glucan synthesis [38]. Given the significant impact of the extracts on *S. mutans* adherence and GTF activity, this suggests that the primary reason for adherence inhibition by the extracts is likely due to GTF inhibition [38]. The ability of plant extracts to disrupt intra- and inter-species quorum sensing (QS) communication systems can act as a defense mechanism against bacterial invasion. The primary mechanisms of action of plant extracts and phytochemicals often involve their chemical similarity to QS signals and their ability to degrade signal receptors [39]. Some studies have demonstrated the protective effects of QS inhibitors against pathogenic bacterial invasion. However, more in vivo research is necessary to obtain conclusive results regarding bacterial pathogenesis and QS inhibition.

The plant phytochemicals play a crucial role in suppressing genes that promote the formation of biofilms and preventing bacterial adhesion. *S. mutans*, a primary cause of dental caries and other oral diseases, can produce firmly bound glucan from sucrose through the cooperative actions of glucosyltransferases (GTFs), which are encoded by the *gtfB*, *gtfC*, and *gtfD* genes [40]. Additionally, it contains the *gbp* gene, which encodes for glucan-binding proteins (GBPs) that facilitate the adherence of the bacteria to surfaces [41]. In this study, the ethanolic extracts of *C. nutans*, *T. laurifolia,* and *A. ebracteatus* could significantly downregulate the *gtfD* gene. The *C. nutans* and *A. ebracteatus* extracts at a concentration of 3.91 mg/mL significantly suppress the mRNA expression of *gbp*. They also inhibit the mRNA expression of the *gtfB* and *gtfC* genes, although this is not a significant difference. This result suggests that the extract could impede the formation of *S. mutans* biofilms by inhibiting its virulence factors. The downregulation of genes, i.e., *gtfD*, would affect the capacity of *S. mutans* to produce α-1,3-linked and α-1,6-linked glucans, as well as fructans, from sucrose [42]. These findings indicate that the plant extracts might reduce the extracellular polysaccharide matrix necessary for biofilm formation. Additionally, the extract inhibits *S. mutans* biofilm formation through regulatory genes and the quorum-sensing cascade. Thus, plant extracts from the Acanthaceae family contain naturally occurring substances with anti-biofilm, antibacterial adhesion, and antibacterial invasion activities. Our findings suggest that plant extracts from the Acanthaceae family prevent the biofilm formation and adhesion of *S. mutans* through two mechanisms: first, by a direct antimicrobial effect, and second, by inhibiting bacterial *gtf* and *gbp* genes, which are considered essential for initial adherence to surfaces, necessary for glucan layer formation, and are considered to promote bacterial accumulation [41,42].

This research offers valuable insights into the potential use of plant extracts from the Acanthaceae family as alternative antibacterial agents, despite their need for higher concentrations compared to conventional antibiotics. Future studies should focus on evaluating the efficacy of the pure compound from these plant extracts at concentrations comparable to those of standard antibiotics. Additionally, exploring the combination of antibiotics with plant-derived compounds that enhance antimicrobial activity could represent a significant advancement in developing novel and effective antimicrobial therapies.

## 4. Materials and Methods

### 4.1. Materials

Plants from the Acanthaceae family, including the leaves of *Thunbergia laurifolia* Lindl. and *Acanthus ebracteatus* Vahl., were purchased from Lanna Herbal Shop, Chiang Mai Province, northern Thailand. The leaves of *Clinacanthus nutans* (Burm.f.) Lindau were collected from Lampang province, northern Thailand. The plant specimens were identified by botanist Dr. Wittaya Pongamornkul and deposited at the Queen Sirikit Botanic Garden Herbarium (QSBG herbarium) under voucher number WP9182. The human squamous carcinoma cell line from tongue (HSC-4) was purchased from the Japanese Collection of Research Bioresources (JCRB) with the accession number JCRB0624.

### 4.2. Reagents and Chemicals

HSC-4 cell culture media and Minimum Essential Medium (MEM) were purchased from Gibco (Grand Island, NY, USA). 3-(4,5-dimethylthizaol-2-yl)-2,5-diphenyl tetrazolium bromide (MTT) was obtained from Bio Basic (Toronto, ON, Canada). Mueller–Hinton (MH) Broth for antimicrobial susceptibility testing was purchased from BD (Becton, Dickinson and Company, Sparks, MD, USA). Chemicals including 2,2-diphenyl-1-picrylhydrazil (DPPH), 2,20-azinobis-(3-ethylbenzothiazolin-6-sulfonic acid) (ABTS), 6-hydroxy-2,5,7,8-tetramethyl-chroman-2-carboxylic acid (Trolox), and gallic acid monohydrate were procured from Sigma-Aldrich (St. Louis, MO, USA). In addition, Folin–Ciocalteu reagent and quercetin dehydrate were sourced from Merck (Billerica, MA, USA).

### 4.3. Medicinal Plant Extraction

**Figure 6:**
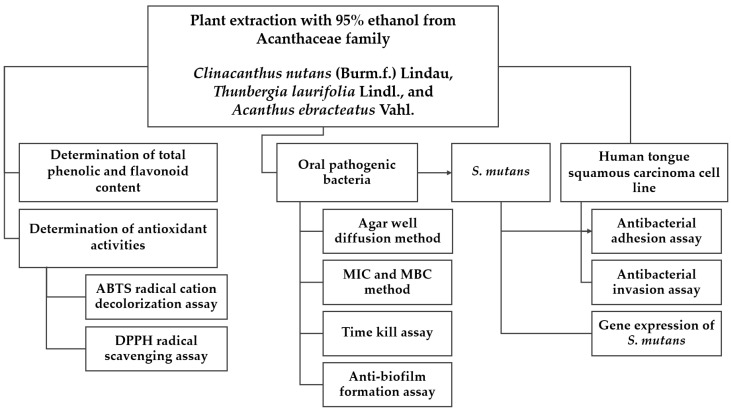
Flowchart of the experimental procedure.

The plant powders from the Acanthaceae family were soaked in 95% ethanol at a ratio of 1:10 (*w*/*v*). Briefly, 200 g of plant powder was mixed with 2000 mL of 95% ethanol. The mixture was orbitally shaken (150 rpm) using a shaker (IKA, Staufen, Germany) at room temperature for 72 h. After this period, the solvent was separated, and the plant powder was re-extracted with an additional 2000 mL of 95% ethanol under the same conditions [43]. The resulting mixture was then filtered through Whatman No.1 filter paper and concentrated using a rotary evaporator (Heidolph, Schwabach, Germany) under vacuum at 45 °C. The concentrated extracts were further dried using freeze-drying (lyophilization) equipment (LABCONCO, Kansas City, MO, USA) and dissolved in dimethyl sulfoxide (DMSO) 99.9% to achieve a concentration of 500 mg/mL for subsequent experimentation. All experiments conducted in this study are summarized in Figure 6, which provides a comprehensive overview of the methodology employed.

### 4.4. Determination of Total Phenolic and Flavonoid Content

The total phenolic content of plant extracts from the Acanthaceae family was measured using the Folin–Ciocalteu method [44]. A mixture of 80 µL of extract, 40 µL of 50% Folin–Ciocalteu reagent, 400 µL of distilled water, 80 µL of 95% ethanol, and 80 µL of 5% *w*/*v* sodium carbonate solution was incubated for 1 h at room temperature. The absorbance was measured at 725 nm using a microplate reader (DYNEX Technologies, Chantilly, VA, USA). The results were expressed as milligrams of gallic acid equivalents (GAE) per gram of extract.

The total flavonoid content was determined by mixing 20 µL of plant extract with 4 µL of 10% aluminum chloride, 60 µL of methanol, 4 µL of 1 M potassium acetate, and 112 µL of distilled water. After a 30 min incubation at room temperature, the absorbance was measured at 415 nm using a microplate reader. The results were expressed as milligrams of quercetin equivalents (QE) per gram of extract [45].

### 4.5. Determination of Antioxidant Activities

The antioxidant activities of plant extracts from the Acanthaceae family were assessed using DPPH and ABTS assays. For the DPPH radical scavenging assay, 50 µL of extract was mixed with 150 µL of 0.1 mM DPPH solution and incubated in the dark at room temperature for 20 min. Absorbance was measured at 517 nm using a microplate reader, and results were expressed as IC_50_ values, with gallic acid as the standard, reported in mg gallic acid equivalents (GAE) per gram of extract [45].

For the ABTS radical cation decolorization assay, 5 µL of plant extract was combined with 195 µL of ABTS solution, adjusted to an absorbance of 0.700 ± 0.020 at 734 nm. After a 10 min incubation, absorbance was measured at 734 nm using a microplate reader. The results were expressed as IC_50_ values, with Trolox as the standard, reported in mg Trolox equivalent antioxidant capacity (TEAC) per gram of extract [46].

### 4.6. Microorganisms

Oral pathogenic bacteria, *Streptococcus pyogenes*, *Streptococcus mutans* ATCC 21575, *Staphylococcus aureus* ATCC 25923, and *Klebsiella pneumoniae* were obtained from SCB 2711 Microbiology Laboratory, Chiang Mai University, and used in this study.

### 4.7. Agar Well Diffusion Method

The effectiveness of plant extracts against bacteria was assessed using the agar well diffusion method. A bacterial suspension was adjusted to the desired turbidity using McFarland standard solution no. 0.5 and then swabbed on brain heart infusion (BHI) agar plates. The medicinal plant extracts were dissolved in BHI broth to obtain final concentrations of 125 mg/mL extracts and 25% DMSO. Wells that were 8 mm in diameter were generated on the agar surface using a sterile cork borer, and 100 µL of extract was added to each well. A standard well containing 1 mg/mL gentamicin and DMSO at 100% served as positive and negative controls, respectively. The plates were then incubated at 37 °C for 24 h under anaerobic conditions, and the antimicrobial activity was determined by measuring the diameter of the inhibition zones against the tested bacterial species [47].

### 4.8. Minimum Inhibitory Concentration (MIC) and Minimum Bactericidal Concentration (MBC)

The minimum inhibitory concentration (MIC) was determined as the lowest concentration of the extracts’ visibly inhibited bacterial growth, assessed through the broth dilution method [47]. Here, 100 µL of plant extracts or standard gentamicin (1 mg/mL) were prepared through serial two-fold dilutions and then added to 96-well plates containing sterile BHI broth. Bacterial inoculum was prepared according to McFarland standard no. 0.5 and then added to the 96-well plates containing the various concentrations of the extracts. The plates were incubated at 37 °C for 24 h under anaerobic conditions, and then examined for growth or turbidity.

The minimum bactericidal concentration (MBC) was assessed by a streak plate procedure carried out on BHI agar plates with samples from 96-well plates lacking observable bacterial growth, followed by incubation at 37 °C for 24 h under anaerobic condition. The MBC endpoint was identified as the lowest concentration of the extracts that eliminated over 99.9% of bacteria [48].

### 4.9. Time–Kill Assay

The time-dependent impact of antimicrobial activity was assessed through a time–kill assay with a few modifications [49]. The plant extracts at a concentration equivalent to 1 MIC were subjected to examination for growth inhibition. Briefly, a bacterial suspension was adjusted to the desired turbidity using McFarland standard solution no. 0.5 and then cultured with the extracts in BHI broth. Bacterial growth was assessed following incubation periods of 0, 2, 4, 6, 8, 10, 12, and 24 h at 37 °C under anaerobic condition. Subsequently, the mixture was diluted with 0.85% saline, and 20 µL of the diluted sample was applied to agar plates using the drop plate technique [50]. After incubation at 37 °C for 24 h under anaerobic conditions, colony counts were determined and compared to the control bacteria without treatment by the extracts.

### 4.10. Anti-Biofilm Formation Assay

Biofilm formation was performed using the crystal violet staining method with some modifications [51]. The bacterial inoculum was adjusted to an initial optical density at 600 nm (OD_600_) of 0.1 by a spectrophotometer (Thermo Scientific, Waltham, MA, USA). The plant extracts at concentrations equivalent to the sub-inhibitory concentration (1/2MIC) were prepared, and concentrations of DMSO in the extracts were 0.782–6.25%. However, the final concentrations of DMSO in the extracts of *C. nutans* and *A. ebracteatus* when tested with *S. pyogenes* were 12% and 25%.

For each concentration, 100 µL was added to individual wells of 96-well plates. Subsequently, 100 µL of bacterial suspension was added to each well of the plates, which were then incubated at 37 °C for 24 h under anaerobic conditions without shaking. After incubation, the planktonic cells were removed, and the wells were washed twice with 1X PBS (pH 7.4). The biofilm was fixed with 95% ethanol for 5 min, after which, the ethanol was discarded. The bacterial biofilm was stained with 0.4% crystal violet for 20 min, and excess dye was washed out twice with 200 µL of phosphate-buffered saline (1X PBS, pH 7.4). The remaining dye was solubilized with 200 µL of 95% ethanol, and the absorbance of the biofilm formation was measured at 592 nm using a microplate reader (DYNEX Technologies, Chantilly, VA, USA). The percentage of inhibition was calculated and compared with the control according to the following equation:

$$Relative\;percentage\;of\;inhibition\;(\%)=\frac{A\;control-A\;treatment}{A\;control}\times 100$$

### 4.11. Cell Culture

The human tongue squamous carcinoma cell line (HSC-4, JCRB0624) was cultured in a growth medium, Minimum Essential Medium (MEM) supplemented with 10% heat-inactivated fetal bovine serum (FBS) and 1% penicillin–streptomycin (100 U/mL penicillin and 100 µg/mL streptomycin), and maintained in a humidified 5% CO_2_ atmosphere at 37 °C using a CO_2_ incubator.

### 4.12. Cell Toxicity Test

Prior to bacteria adherence to and invasion of the human tongue squamous cell carcinoma-derived cell line (HSC-4 cells), the potent toxicity of extracts on HSC-4 cells was assessed in vitro using a 3-(4,5-dimethylthiazol-2-yl)-2,5 diphenyltetrazolium bromide (MTT) assay. Briefly, HSC-4 cells were cultured in 96-well plates at a density of 1 × 10^6^ cells/mL and incubated at 37 °C in a 5% CO_2_ incubator for 24 h. Subsequently, the culture media were removed, and the cells were treated with 100 µL of either the control medium or medium containing different concentrations of the extracts (19.53–2500 µg/mL). DMSO at final concentrations of 0.062–0.12% was used to dissolve the extracts. Then, the cells were further incubated at 37° for 24 h. MTT solution (2 mg/mL in 1X PBS) was added to each well, followed by a 4 h incubation at 37 °C. After incubation, the medium was removed, and DMSO (200 μL) was added to solubilize the formed formazan crystals [52]. The absorbance was measured on a microplate reader (DYNEX Technologies, Chantilly, VA, USA) using a tested wavelength of 540 nm and a reference wavelength of 630 nm. Non-toxic concentrations of extracts, which did not significantly affect the cell viability of HSC-4 cells compared to the cell control group, were selected to study their antibacterial adhesion and invasion activities. These concentrations of plant extracts from the Acanthaceae family were used as follows: *C. nutans* and *T. laurifolia* at a concentration of 0.63 mg/mL and *A. ebracteatus* at a concentration of 0.31 mg/mL.

### 4.13. Antibacterial Adhesion Assay

The cells were cultured on microscope cover glasses within 24-well plates at a density of 1 × 10^4^ cells/mL and incubated at 37 °C in a 5% CO_2_ incubator for 24 h. Subsequently, the media were removed, and the cells were infected with 200 µL of bacterial suspension of *S. mutans*, which was adjusted to 1 × 10^8^ CFU/mL and treated with 200 µL of plant extracts. After incubation for 3 h, the supernatants were removed, and the cells were washed 2–3 times with 1X PBS (pH 7.4). The cells were then fixed with methanol for 5 min and stained with 0.38% Giemsa for 15 min [53]. After excess stain removal, the microscope cover glasses were investigated under a light microscope (OLYMPUS CX31, Center Valley, PA, USA). The percentage of antibacterial adhesion was calculated by counting the number of cells to which bacteria had adhered and compared to the negative control using the following formula:

$$Percentage\;of\;antibacterial\;adhesion\;(\%)=\frac{N\;control-N\;treatment}{N\;control}\times 100$$

### 4.14. Antibacterial Invasion Assay

The cells were cultured on microscope cover glasses within 24-well plates at a density of 1 × 10^5^ cells/mL and incubated at 37 °C in a 5% CO_2_ incubator for 24 h. Subsequently, the media were removed, and the cells were infected with 200 µL of bacterial suspension of *S. mutans* adjusted to 1 × 10^8^ CFU/mL and treated with 200 µL of plant extracts. After incubation for 3 h, the supernatants were discarded from the wells. The cells were washed 2–3 times with 1X PBS (pH 7.4), and then, 100 µg/mL of gentamicin was added to the cells and incubated for 30 min. After washing the cells 2–3 times with 1X PBS (pH 7.4), 1% Triton X-100 was added to lyse the cell for 5 min. A 100 µL aliquot of each sample was then diluted in 1X PBS (pH 7.4). The diluted sample (100 µL) was spread onto BHI agar plates and incubated at 37 °C for 24 h [37]. Colony counts of intracellular bacteria were evaluated and compared to the negative control using the following formula:

$$Percentage\;of\;antibacterial\;invasion\;(\%)=\frac{N\;control-N\;treatment}{N\;control}\times 100$$

### 4.15. Effect of the Plant Extracts from the Acanthaceae Family on Gene Expression of S. mutans

#### 4.15.1. RNA Extraction and cDNA Synthesis

*S. mutans* were treated with the extracts at concentrations of 3.91 and 1.95 mg/mL and incubated at 37 °C for 24 h under anaerobic conditions. Subsequently, the bacterial suspension was centrifuged at 6000 rpm for 10 min. The bacterial cell pellet was then washed with 1X PBS (pH 7.4) twice through centrifugation at 6000 rpm for 5 min. Total RNA was then isolated from the bacterial cell pellet using Trizol^®^ reagent (Invitrogen, Carlsbad, CA, USA) according to the manufacturer’s instructions. Next, the total RNA was converted into cDNA using a reverse transcription system comprising 2 µL of 5× RT Master MIX (ReverTra Ace^®^ TOYOBO, Osaka, Japan), 2 µg of total RNA, and 6 µL of RNase-free water. The reaction proceeded at 37 °C for 15 min, followed by incubation at 50 °C for 5 min and then heating at 98 °C for 5 min.

#### 4.15.2. Real-Time Quantitative PCR Amplification

The cDNA was used for real-time qPCR amplification using the SensiFAST™ SYBR^®^ No-ROX Kit (BIOLINE, London, UK) to analyze genes of *S. mutans* (*gtfB*, *gtfC*, *gtfD*, *gbp*). This procedure was conducted in accordance with the instructions provided in the SensiFAST SYBR^®^ No-ROX Kit manual. The qPCR reaction mixture comprised 10 µL of SensiFAST SYBR^®^ (2×), 0.8 µL of forward primer (10 µM), 0.8 µL of reverse primer (10 µM), and 8.4 µL of cDNA, under the following reaction conditions: 95.0 °C for 2 min, followed by 40 cycles at 95.0 °C for 5 s and 65.0 °C for 30 s. The resulting amplification data were presented as the threshold cycle (Ct) value, indicating the number of cycles required to generate a fluorescent signal greater than a predefined threshold. The expression levels of virulence genes were normalized using 16S rRNA as an internal control. The primers targeting genes are listed in Table 7.

### 4.16. Statistical Analysis

The statistical analysis was performed using SPSS 17.0 software (IBM, Chicago, IL, USA). The quantitative data were verified in triplicate and reported as mean ± standard deviations. A one-way analysis of variance (ANOVA) was carried out, followed by Tukey’s honestly significant difference (HSD) post hoc test to ascertain the significance among all groups, with *p*-values below 0.05 (*p* < 0.05) considered statistically significant.

## 5. Conclusions

In this study, we demonstrated that plant extracts from the Acanthaceae family are rich in phenolics and flavonoids, which significantly contribute to their antioxidant activity and antimicrobial activity against some oral pathogenic bacteria. This preliminary investigation focused on three plant species: *Clinacanthus nutans* (Burm.f.) Lindau, *Thunbergia laurifolia* Lindl., and *Acanthus ebracteatus* Vahl. The results provided valuable information about the inhibitory effects of these extracts on oral pathogenic bacteria. The insights from these data could lead to the discovery of new, effective antimicrobial agents derived from natural sources, contributing to the advancement of treatments for bacterial infections and improving public health outcomes. However, further investigation, including experimental models and pharmacological evaluations, are necessary before considering these plant extracts as truly promising compounds.

## Figures and Tables

**Table 1:** Total phenolic and flavonoid contents of plant extracts from the Acanthaceae family.

Plant Extracts	Total Phenolic Content (mg GAE/g Extract)	Total Flavonoid Content (mg QE/g Extract)
*C. nutans*	1.86 ± 0.31 ^a^	14.12 ± 0.52 ^b^
*T. laurifolia*	9.87 ± 0.33 ^b^	3.29 ± 0.24 ^a^
*A. ebracteatus*	22.55 ± 0.48 ^c^	4.07 ± 0.49 ^a^

Each value in the table represents the mean ± standard deviation (n = 3). Statistical significance was denoted by different letters (^a–c^) indicating a significant difference (*p <* 0.05). The statistical analysis was conducted using ANOVA followed by post hoc Tukey’s test.

**Table 2:** Assessment of antioxidant activities in plant extracts from the Acanthaceae family using DPPH and ABTS assays.

Plant Extracts	DPPH		ABTS	
	IC_50_ (mg/mL)	Antioxidant Activity (mg GAE/g Extract)	IC_50_ (mg/mL)	Antioxidant Activity (mg TEAC/g Extract)
*C. nutans*	0.84 ± 0.02 ^b^	6.75 ± 0.14 ^a^	6.10 ± 0.39 ^c^	28.67 ± 1.46 ^a^
*T. laurifolia*	0.62 ± 0.06 ^ab^	9.17 ± 0.84 ^a^	4.41 ± 0.19 ^b^	42.47 ± 2.22 ^b^
*A. ebracteatus*	0.24 ± 0.06 ^a^	24.41 ± 6.19 ^b^	3.05 ± 0.31 ^a^	57.47 ± 4.91 ^c^

Each value in the table represents the mean ± standard deviation (n = 3). Statistical significance was denoted by different letters (^a–c^) indicating a significant difference (*p* < 0.05). The statistical analysis was conducted using ANOVA followed by post hoc Tukey’s test.

**Table 3:** Antibacterial activity of plant extracts from the Acanthaceae family against some oral pathogenic bacteria.

Plant Extracts	Inhibition Zone Diameter (mm)			
	*K. pneumoniae*	*S. aureus*	*S. mutans*	*S. pyogenes*
*C. nutans*	0 ^a^	0 ^a^	0 ^a^	17.7 ± 1.5 ^c^
*T. laurifolia*	0 ^a^	14.7 ± 0.6 ^b^	16.3 ± 1.5 ^c^	17.3 ± 1.2 ^c^
*A. ebracteatus*	0 ^a^	0 ^a^	0 ^a^	0 ^a^
Gentamicin (1 mg/mL)	24.3 ± 0.6 ^d^	27.3 ± 0.6 ^d^	24.7 ± 1.2 ^d^	26.0 ± 1.0 ^d^
DMSO 99.9%	0 ^a^	0 ^a^	0 ^a^	0 ^a^

Each value in the table represents the mean ± standard deviation (n = 3) using a plant extract concentration of 125 mg/mL. The results show the diameter (in mm) of the inhibition zone determined by the well diffusion method. Statistical significance was indicated by different letters (^a–d^) representing a significant difference (*p* < 0.05). The statistical analysis was conducted using ANOVA followed by post hoc Tukey’s test. Gentamicin at a concentration of 1 mg/mL served as the positive control and DMSO 99.9% served as the negative control.

**Table 4:** Antibacterial activity of plant extracts from the Acanthaceae family as determined by minimum inhibitory concentration (MIC) and minimum bactericidal concentration (MBC).

Plant Extracts	Concentration of Plant Extract (mg/mL)							
	*K. pneumoniae*		*S. aureus*		*S. mutans*		*S. pyogenes*	
	MIC	MBC	MIC	MBC	MIC	MBC	MIC	MBC
*C. nutans*	62.5	62.5	62.5	62.5	15.63	15.63	125	125
*T. laurifolia*	62.5	62.5	15.63	15.63	15.63	15.63	7.81	7.81
*A. ebracteatus*	62.5	62.5	31.25	31.25	62.5	62.5	250	250
Gentamicin (1 mg/mL)	0.031	0.031	0.0156	0.0156	0.063	0.125	0.0039	0.0156

**Table 5:** Inhibition of biofilm formation of plant extracts from the Acanthaceae family against some oral pathogenic bacteria.

Plant Extracts	% Inhibition of Biofilm Formation			
	*K. pneumoniae*	*S. aureus*	*S. mutans*	*S. pyogenes*
*C. nutans*	88.89 ± 4.32 ^cd^	92.49 ± 5.10 ^d^	37.95 ± 3.68 ^a^	95.12 ± 2.94 ^d^
*T. laurifolia*	75.40 ± 8.56 ^c^	84.79 ± 7.67 ^c^	73.38 ± 8.34 ^c^	34.22 ± 8.92 ^a^
*A. ebracteatus*	63.63 ± 5.40 ^b^	42.51 ± 8.69 ^a^	95.45 ± 5.23 ^d^	96.84 ± 2.69 ^d^

Each value in the table represents the mean ± standard deviation (n = 3). Statistical significance was denoted by different letters (^a–d^) indicating a significant difference (*p* < 0.05). The statistical analysis was conducted using ANOVA followed by post hoc Tukey’s test.

**Table 6:** Effect of plant extracts from the Acanthaceae family on antibacterial adhesion and invasion of *S. mutans* to HSC-4 cells.

Plant Extracts	% Inhibition of Bacterial Adhesion and Invasion	
	Bacterial Adhesion	Bacterial Invasion
*C. nutans*	42.03 ± 9.31 ^ab^	80.40 ± 6.91 ^c^
*T. laurifolia*	48.97 ± 0.44 ^b^	70.83 ± 7.98 ^c^
*A. ebracteatus*	22.47 ± 2.44 ^a^	80.97 ± 3.02 ^c^

Each value in the table represents the mean ± standard deviation (n = 3). Statistical significance was denoted by different letters (^a–c^) indicating a significant difference (*p* < 0.05). The statistical analysis was conducted using ANOVA followed by post hoc Tukey’s test.

**Table 7:** The primers of *S. mutans* used for real-time qPCR analysis.

Genes	Sense Primer Sequence 5′-3′	Antisense Primer Sequence 5′-3′
*gtfB*	GCACCCCGACCAATCAAACT	GCCTGCACGACAGGATTAGA
*gtfC*	CGCACCCCGACTAATCAAAC	GTGGAGCCAGTTCAGCTGTT
*gtfD*	GGCAAAACGTGGACAGCTT	GTTCCAAGCCCTTGCTGGT
*gbp*	CTGGAGAAGCTCAGTCAGTGC	GAAGCTATTGGTTGGAGCAGC
16s rRNA	CATGTGTAGCGGTGAAATGCG	CTCATCGTTTACGGCGTGGAC

## Data Availability

The original contributions presented in the study are included in the article/Supplementary Materials; further inquiries can be directed to the corresponding author.

## Supplementary Materials

The following supporting information can be downloaded at https://www.mdpi.com/article/10.3390/plants13182622/s1: Table S1: Time–kill assay.

## Abbreviations

ABTS: 2,20-azinobis-(3-ethylbenzothiazolin-6-sulfonic acid); BHI: brain heart infusion; DMSO: dimethyl sulfoxide; DPPH: 2,2-diphenyl-2-picrylhydrazyl hydrate; GAE: gallic acid equivalents; GTFs: glucosyltransferases; GBPs: glucan-binding proteins; IC_50_: 50% inhibitory concentration; MEM: minimum essential medium; HSC-4: human tongue squamous carcinoma cell; MH: Mueller–Hinton; MIC: minimum inhibitory concentration; MBC: minimum bactericidal concentration; MTT: 3-(4,5-dimethylthizaol-2-yl)-2,5-diphenyl tetrazolium bromide; PIA/PNAG: polysaccharide intercellular adhesin/poly-N-acetylglucosamine; QE: quercetin equivalents; qPCR: quantitative polymerase chain reaction; QS: quorum sensing; SarA: staphylococcal accessory regulator A; TEAC: Trolox equivalent antioxidant capacity; *w/v*: weight/volume.
